# FGF4 induces epithelial-mesenchymal transition by inducing store-operated calcium entry in lung adenocarcinoma

**DOI:** 10.18632/oncotarget.12187

**Published:** 2016-09-22

**Authors:** Lisha Qi, Wangzhao Song, Lingmei Li, Lu Cao, Yue Yu, Chunmin Song, Yalei Wang, Fei Zhang, Yang Li, Bin Zhang, Wenfeng Cao

**Affiliations:** ^1^ Department of Pathology, Tianjin Medical University Cancer Institute and Hospital, Tianjin 300060, China; ^2^ Department of Breast Cancer, Tianjin Medical University Cancer Institute and Hospital, Tianjin 300060, China; ^3^ Department of Pancreatic Cancer, Tianjin Medical University Cancer Institute and Hospital, Tianjin 300060, China; ^4^ Tianjin Medical University, Tianjin 300070, China; ^5^ The Key Laboratory of Tianjin Cancer Prevention and Treatment, Tianjin 300060, China; ^6^ National Clinical Research Center for Cancer, Tianjin 300060, China; ^7^ Research Center of Basic Medical Sciences, Tianjin Medical University Cancer Institute and Hospital, Tianjin 300060, China; ^8^ Department of Family Planning, Maternity & Child Care Center of Luoyang, Luoyang 471000, China

**Keywords:** FGF4, epithelial-mesenchymal transition, store-operated calcium entry, Orai1, FGF7

## Abstract

Several fibroblast growth factor (FGF) isoforms act to stimulate epithelial-mesenchymal transition (EMT) during cancer progression. FGF4 and FGF7 are two ligands of FGF receptor 2 (FGFR2). Using two lung adenocarcinoma (ADC) cell lines, A549 and H1299, we showed that FGF4, but not FGF7, altered cell morphology, promoted EMT-associated protein expression, and enhanced cell proliferation, migration/invasion and colony initiation. In addition, FGF4 increased store-operated calcium entry (SOCE) and expression of the calcium signal-associated protein Orai1. The SOCE inhibitor 2,5-di-tert-butylhydroquinone (BHQ) or Orai1 knockdown reversed all of the EMT-promoting effects of FGF4. BHQ also inhibited FGF4-induced EMT in a mouse xenograft model. Finally, 60 human lung ADC samples and 21 sets of matched specimens (primary and metastatic foci in lymph nodes from one patient) were used to confirm the clinicopathologic significance of FGF4 and its correlation with E-cadherin, Vimentin and Orai1 expression. Our study thus shows that FGF4 induces EMT by elevating SOCE in lung ADC.

## INTRODUCTION

The fibroblast growth factor (FGF) family consists of at least 23 members. FGFs are involved in numerous physiological processes, ranging from vertebrate segmentation and elongation of the embryonic axis to skeletal development [1, 2]. FGF overexpression has been linked with the pathogenesis and progression of multiple cancers, including breast, prostate [3] and lung cancer [4].

Epithelial-mesenchymal transition (EMT) is a crucial process in cancer progression. During EMT, epithelial cells escape from their origin, gain fibroblastic characteristics, and migrate to distant locations [5, 6]. The EMT program is promoted by many soluble growth factors, including FGFs [2, 7]. For example, the activation of the FGFR/MEK/ERK pathway by FGF2 promotes cell growth and EMT in the chordoma [8] and FGF10 can induce EMT of breast cancer cells [9]. Furthermore, FGF8 can induce a more aggressive phenotype displaying EMT and enhanced invasion and growth in colorectal cancer cells [10]. Takanami *et al*. measured FGF2 expression in 143 lung adenocarcinoma (ADC) tissues [11] and found that high FGF2 expression was a prognostic indicator for unfavorable outcome. Donnem and colleagues showed that high FGF2 expression in non-small-cell lung cancer (NSCLC) is associated with poor five-year survival [4]. Li demonstrated that immunoreactive scores of FGF1 were higher in NSCLC specimens than in peritumoral normal tissues and patients with high FGF1 expression had a lower overall survival rate [12]. NSCLC patients with high FGF9 expression were also reported to have a worse prognosis than those with low FGF9 expression [13].

FGF signals are transduced through FGF receptors (FGFRs). Alternative splicing of FGFR2 mRNA generates the FGFR2-IIIb isoform, which selectively binds FGF7 with high affinity, or the FGFR2-IIIc isoform, which selectively binds FGF4. The dedicated ligands for FGFR2-IIIb and FGFR2-IIIc are usually expressed in mesenchymal and epithelial cells, respectively [2, 14, 15]. A switch from FGFR2-IIIb to FGFR2-IIIc has been reported in the malignant progression of prostate and bladder cancer [14, 16]. In our study, out of the 23 reported FGFs, we focused on FGF4 and FGF7, the representative FGF ligands for FGFR2 splice variants, and studied their functions in the EMT program of lung ADC.

EMT has been linked with the dysregulation of FGF/FGFR signaling, including MEK/ERK and PI3K-AKT signaling [2, 8, 17]. In addition, the activation of FGF/FGFR can activate inositol trisphosphate (IP3), which triggers Ca^2+^ signaling [18]. As a ubiquitous secondary messenger, intracellular calcium regulates nearly every aspect of cellular function, including excitability, exocytosis, cytoskeletal reorganization, motility, deformability, and gene expression [19]. Altered Ca^2+^ homeostasis is also a hallmark of numerous cancers, including breast, prostate, and ovarian cancers [6, 20-23]. Store-operated calcium entry (SOCE) is responsible for activating Ca^2+^ channels in the plasma membrane following depletion of Ca^2+^ stores. SOCE is the foremost pattern for ectocytic Ca^2+^ entry in non-excitable epithelial cells [19, 24, 25]. The molecular components of SOCE include the endoplasmic reticulum Ca^2+^ sensors stromal interaction molecule1 (STIM1), STIM2 and the store-operated Ca^2+^ channel-forming subunits Orai1, Orai2, and Orai3. Orai1 is recognized as the central component of SOCE because of it exhibits the highest potency in the conduction of Ca^2+^ currents [25, 26]. Although several studies reported that altered SOCE is linked to EGF [22] and TGF-induced EMT [27], the effects of SOCE induced by FGF in EMT remain unclear.

In the present study, we show that FGF4, but not FGF7, induces EMT and promotes proliferation, migration/invasion, and colony initiation in A549 and H1299 lung ADC cell lines. More importantly, we demonstrate that FGF4 induces EMT via enhancement of SOCE in lung ADC.

## RESULTS

### Treatment with FGF4, but not FGF7, induces EMT, causes a switch from *FGFR2 IIIb* to *FGFR2 IIIc* and changes the morphology and behavior of A549 and H1299 cells

**Figure 1:**
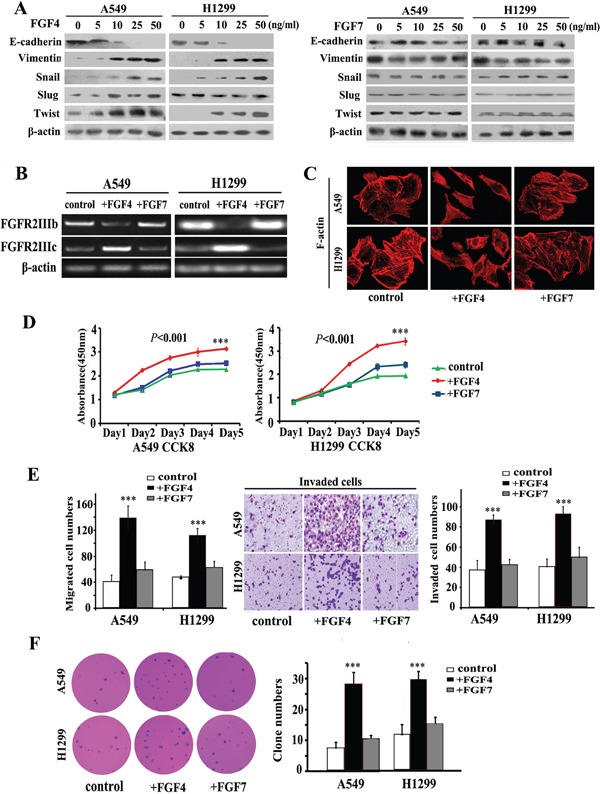
FGF4, not FGF7 treatment induces EMT, causes a switch from FGFR2 IIIb to FGFR2 IIIc and changes the cell morphology and behavior of A549 and H1299 lung ADC cells **A.** Western blot assay measuring the expression of epithelial protein (E-cadherin), mesenchymal protein (Vimentin), and EMT transcription factors (Snail, Slug, and Twist) in A549 and H1299 cells treated with FGF4 and FGF7 at different concentrations of 0, 5, 10, and 25, and 50 ng/mL, respectively. **B.** RT-PCR was performed to detect gene expression alterations in *FGFR2 IIIb* and *FGFR2 IIIc*. **C.** Cell morphology was analyzed by confocal laser scanning microscopy according to the immunolocalization of F-actin. **D.** CCK-8 cell proliferation assay was conducted in cells upon incubation with FGF4 or FGF7 in 1, 2, 3, 4, and 5 d, respectively. **E.** Transwell assay showing migration/invasion of cells treated with FGF4 or FGF7. **F.** Anchorage-independent growth was conducted to show the colony-initiation ability of cells exposed to FGF4 or FGF7. A549 and H1299 cells were treated with FGF4 and FGF7 at 10 ng/mL for 24 h, respectively. The control is A549 and H1299 cells without FGF4 or FGF7 stimulation. All graphs represent the mean ± SD of three independent experiments. The axis represents the fold change in the number of cells. ***P < 0.001.

To evaluate the effect of FGF4 and FGF7 on promoting EMT, A549 and H1299 cells were treated at different concentrations of 0, 5, 10, 25, and 50 ng/mL for 24 h. As shown in Figure 1A, FGF4 decreased E-cadherin and increased Vimentin expression in both cell lines. In addition to the classical EMT markers, we also checked the expression of an array of EMT transcription factors, including Snail, Slug, and Twist. These factors could induce EMT by directly binding to the E-boxes of the E-cadherin promoter. Among these three factors, Snail and Twist were upregulated in the cells exposed to FGF4. Moreover, the EMT-promoting effect of FGF4 on both cells was dose-dependent. In contrast to FGF4, FGF7 showed no evident influence on the expression of these EMT-associated proteins.

Except for alterations in epithelial and mesenchymal proteins expression, we also found alterations in cellular morphology and functional phenotypes. By using phalloidin to dye fibrous actin (F-actin), we observed that FGF4 treatment (10 ng/m, 24 h) caused A549 and H1299 cells to form structures with irregular shape and non-uniform composition (Figure 1C). In CCK8 assays, we observed that FGF4 could promote cell proliferation as early as the third day (Figure 1D). In migration and invasion assays, significantly more cells passed through the transwell chamber filter when stimulated with FGF4, compared with control cells (Figure 1E). Anchorage-independent growth was increased in cells with FGF4 stimulation (Figure 1F). Compared with control cells, FGF7 showed no influence on proliferation, migration/invasion, and colony-initiating ability of A549 and H1299 cells.

### FGF4 elevates intracellular calcium concentration and increases the expression of Orai1

**Figure 2:**
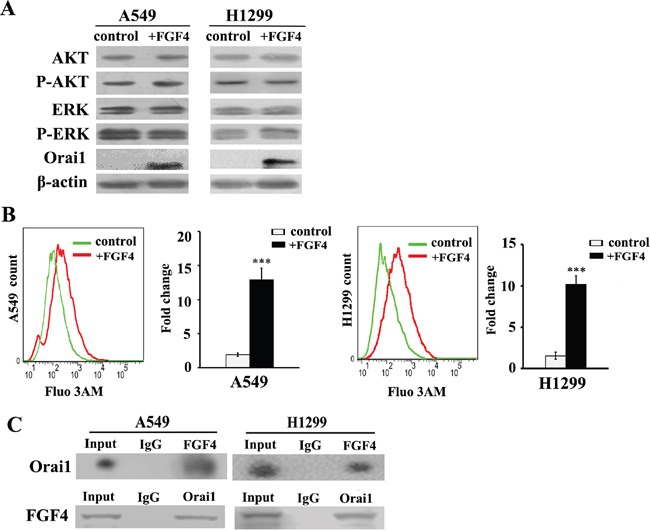
FGF4 elevates intracellular calcium concentration and upregulates the expression of Orai1 A549 and H1299 cells were treated with FGF4 at 10 ng/mL for 24 h. A549 and H1299 cells without FGF4 stimulation were used as controls. **A.** Western blot of AKT, ERK, phosphorylated AKT, phosphorylated ERK and Orai1 in A549 and H1299 cells after treatment with FGF4 for 20 minutes. **B.** Changes in the level of intracellular Ca^2+^ were measured by flow cytometry. **C.** Reciprocal co-immunoprecipitation of endogenous FGF4 and Orai1 in A549 and H1299 cells. All graphs represent the mean ± SD of three independent experiments. The axis represents the fold change in the number of cells. ***P < 0.001.

Binding of some FGF ligands to FGFRs induces activation of the downstream intracellular signaling components including MEK/ERK and PI3K-AKT. However, FGF4 stimulation did not alter the expression of AKT, ERK and phosphorylated AKT and ERK, which are key signal transducers downstream of the FGFR (Figure 2A). FGF/FGFR signaling also evokes Ca^2+^ release from calcium stores into the cytosol, which reportedly contributes to the EMT process. We detected Orai1 expression and found that Orai1 was significantly upregulated after FGF4 treatment in both cell lines (Figure 2A). We further evaluated intracellular calcium concentration by Fluo 3-AM as a calcium indicator. Our flow cytometry experiments demonstrated that the mean fluorescence intensity (MFI) of the FGF4 group (10.16 × 10^3^) was significantly higher than the MFI of the control (1.5 × 10^3^) (*P* < 0.01) (Figure 2B). The interaction between FGF4 and Orai1 was further confirmed by reciprocal co-IP analysis (Figure 2C). Furthermore, NVP-BGJ398 (a selective inhibitor of the FGFR) could restore intracellular calcium concentration and abolish the expression of Orai1 caused by FGF4 stimulation (Supplementary Figure S1), demonstrating the SOCE-elevating effect of FGF4/FGFR signaling.

### Orai1 knockdown and BHQ impair FGF4-induced EMT in A549 and H1299 cells

**Figure 3:**
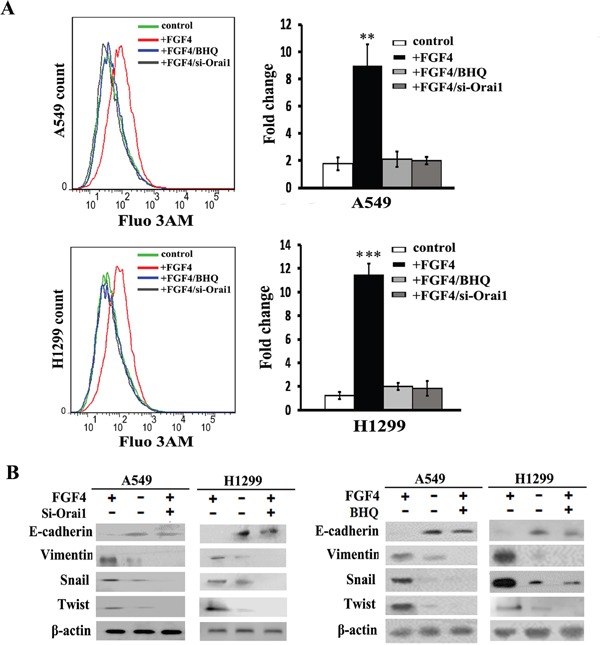
Orai1 knockdown and BHQ impair FGF4-induced EMT in A549 and H1299 cells A549 and H1299 cells were transfected with Orai1 siRNA for 24 h or stimulated with BHQ (50 μM) for 12 h and then treated with FGF4 (10 ng/mL) for 24 h. A549 and H1299 cells transfected with non-sense control siRNA and without FGF4 or BHQ treatment were used as controls. **A.** The level of intracellular Ca^2+^ using Fluo 3-AM was measured by flow cytometry. **B.** The expression of EMT-associated proteins (E-cadherin, Vimentin, Snail and Twist) was detected by western blot. All graphs represent the mean ± SD of three independent experiments. The axis represents the fold change in the number of cells. **P < 0.01, ***P < 0.001.

Given that SOCE is the predominant pattern for extracellular Ca^2+^ influx in non-excitable cells, we investigated whether SOCE contributes to the EMT response to FGF4. A549 and H1299 cells were transfected with Orai1 siRNA (si-1, Supplementary Figure S2) for 24 h, and then treated with 10 ng/ml FGF4. BHQ is an inhibitor of the smooth endoplasmic reticulum (Ca^2+^-Mg^2+^) ATPase and functions as an endoplasmic reticulum Ca^2+^ pump inhibitor. We observed that both Orai1 knockdown and BHQ could reverse the increased cytosolic Ca^2+^ concentration caused by FGF4 (Figure 3A), indicating that Orai1 and BHQ have an inhibitory effect on Ca^2+^ signaling. As shown in Figure 3B, all mesenchymal factors (Vimentin, Snail and Twist) increased due to FGF4 stimulation with contrary expression of epithelial marker (E-cadherin). Western blot showed that either Orai1 knockdown or BHQ treatment could rescue E-cadherin inhibition and decrease Vimentin, Snail and Twist levels induced by FGF4 (Figure 3B), suggesting that SOCE was involved in FGF4-induced EMT. To rule out the different Si-Orai1 variations, we also transfected another Orai1 siRNA (Si-2) into A549 and H1299 cells and tested the expression of EMT-associated proteins by western blot (Supplementary Figure S3). The results were similar to those obtained from Orai1 siRNA (si-1).

### BHQ and orai1 knockdown induce restoration of epithelial phenotype, impair migration and invasion, and inhibit cell growth in monolayer cultures and anchorage-independent growth in soft agar of A549 and H1299 cells

**Figure 4:**
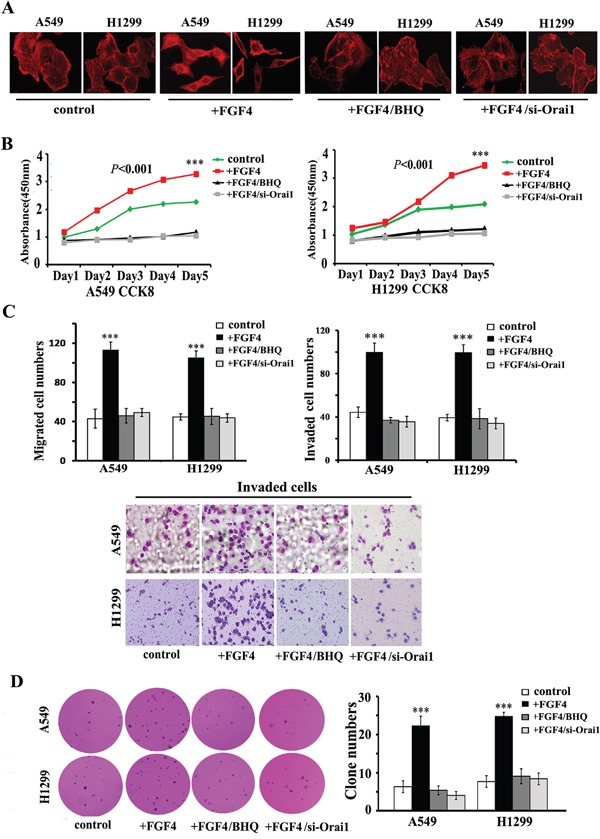
BHQ and Orai1 knockdown induce restoration of epithelial phenotype, impair migration and invasion, inhibit cell growth in monolayer cultures and anchorage-independent growth in soft agar of A549 and H1299 cells **A.** Confocal laser scanning microscopy according to the immunolocalization of F-actin, **B.** CCK-8 cell proliferation assay, **C.** transwell assays and **D.** anchorage-independent growth assay of A549 and H1299 cells transfected with Orai1 siRNA for 24 h or stimulated with BHQ (50 μM) for 12 h and then treated with FGF4 (10 ng/mL) for 24 h. A549 and H1299 cells transfected with non-sense control siRNA and without FGF4 or BHQ treatment were used as controls. All graphs represent the mean ± SD of three independent experiments. The axis represents the fold change in the number of cells. ***P < 0.001.

We next assessed whether FGF4-induced mesenchymal cell features could be blocked by SOCE inhibition. As expected, either BHQ or Orai1 knockdown changed the morphology of A549 and H1299 cells from a spindle-like shape to a cobble stone-like shape (Figure 4A), inhibited cell growth in monolayer cultures (Figure 4B), migration/invasion (Figure 4C), and colony-initiation (Figure 4D) in A549 and H1299 cells with FGF4 treatment.

### BHQ inhibits *in vivo* tumor growth and metastasis caused by FGF4 in a H1299 xenograft mouse model, induces E-cadherin expression, and decreases the expression of Orai1 and Vimentin in tumor tissues

**Figure 5:**
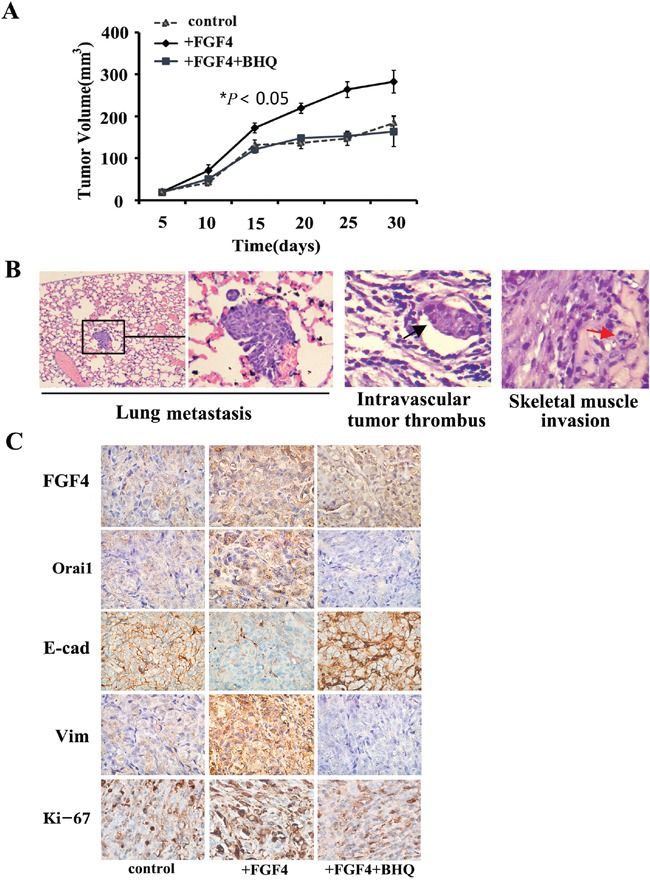
BHQ inhibits *in vivo* tumor growth and metastasis caused by FGF4 in a H1299 xenograft mouse model, induces the expression of E-cadherin, and decreases the expression of Orai1 and Vimentin in tumor tissues **A.** H1299 cells with FGF4 treatment grew into larger tumor masses than the control cells and the cells with FGF4+BHQ treatment. **B.** Mice injected with H1299 cells with FGF4 stimulation showed lung metastasis, intravascular thrombus and skeletal muscle invasion. H&E staining, (200×). **C.** Immunohistochemical staining of FGF4, Orai1, E-cadherin Vimentin and Ki-67 expression in harvested H1299 xenograft mouse samples. 200×. All graphs represent mean ± SD of three independent experiments. *P < 0.05.

Using the highly metastatic H1299 lung ADC cells, we made a mouse xenograft model to assess the effect of BHQ on tumor growth and metastasis *in vivo*. In accordance with our *in vitro* findings, cells with FGF4 treatment grew into larger tumor masses than control cells and cells with FGF4+BHQ treatment (Figure 5A). Of the 10 mice injected with cells with FGF4 stimulation, two showed lung metastasis, two showed intravascular thrombus and three showed tumor invasion into skeletal muscle (Figure 5B), whereas no metastasis sites were detected in mice injected with control cells and cells with FGF4+BHQ treatment. We then performed FGF4, Orai1, E-cadherin, Vimentin and Ki-67 immunohistochemical staining on the sections of xenograft tissues. Immunohistochemical analyses showed that tumor sections from FGF4-stimulated cells exhibited a marked increase in Orai1 whereas tumor sections from control and FGF4+BHQ-stimulated cells revealed no or weak staining. Moreover, compared with control cells, FGF4-treated cells showed decreased E-cadherin expression and increased Vimentin and Ki-67 expression. BHQ reversed FGF4-induced expression of EMT-associated proteins, including E-cadherin and Vimentin (Figure 5C). These results substantiate our findings that FGF4 induces SOCE enhancement, which contributes to promoting the EMT process in lung ADC cells.

### Association of FGF4 expression with clinicopathological features of lung ADC cases

**Figure 6:**
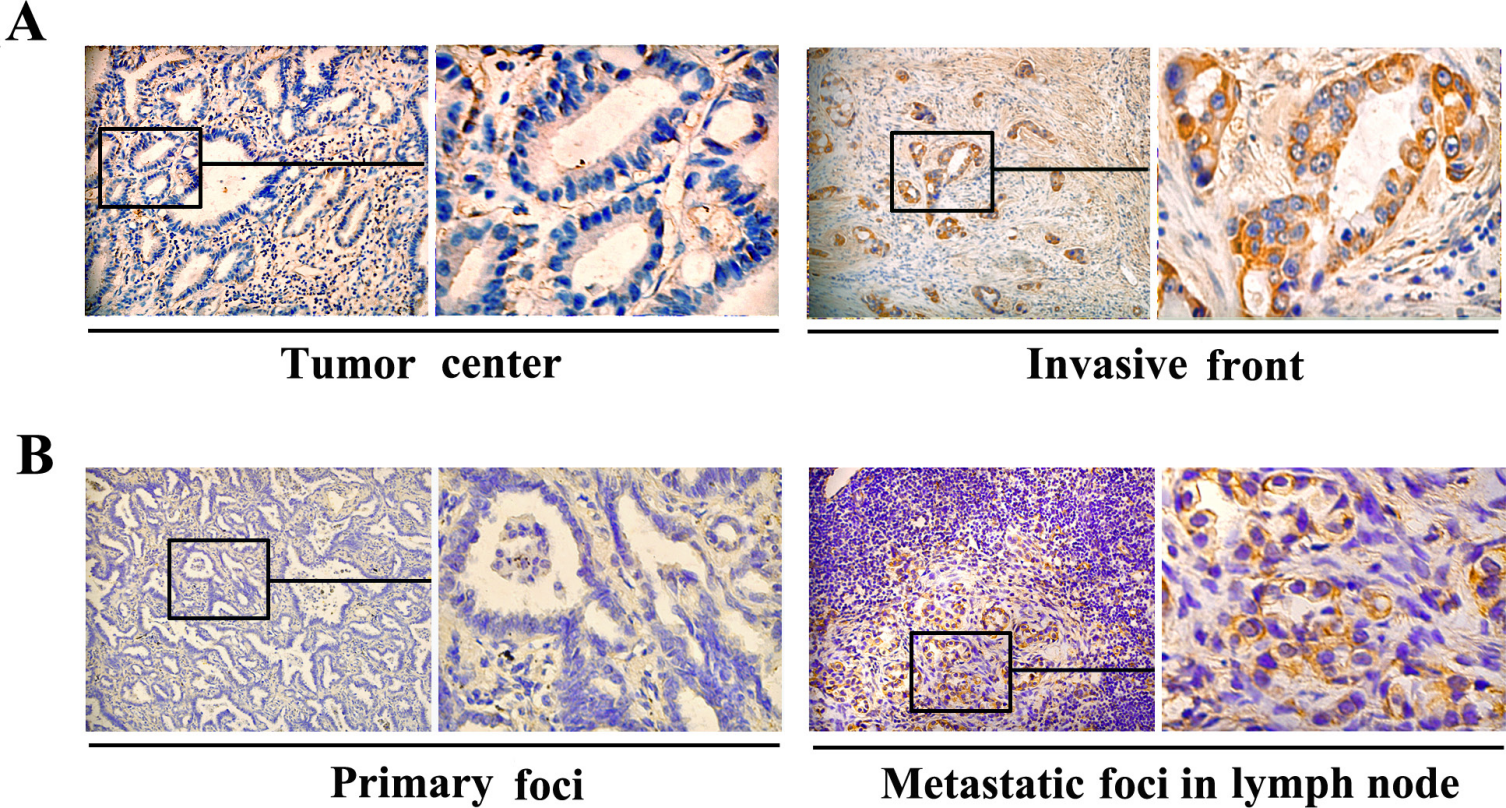
Differences in FGF4 levels within human ADC tissues and in matched lung ADC primary foci and lymph node metastatic foci Immunohistochemical staining of FGF4 showed **A.** different FGF4 levels in the tumor center and the invasive front of the same lung ADC tissue sample (200×), **B.** different FGF4 levels inlung ADC primary foci and lymph node metastatic foci from a same patient (200×).

The expression patterns of FGF4 were examined on an array of 60 human lung ADC cases. Among 60 samples, 38 (63.3%) showed positive FGF4 expression. Tumors were categorized as strong (++), weak (+), or negative (−) expression for FGF4. Relationships between FGF4 levels and each clinicopathological parameter are summarized in Table 1. FGF4 expression was strongly correlated with histological type (*P* < 0.05), TNM stages (*P* < 0.05), and metastasis (*P* < 0.05). The frequency of positive FGF4 expression was higher in solid predominant and micropapillary predominant samples than in ones with other histological types. Positive FGF4 expression was observed in 11 of 13 patients (84.6%) with advanced stage carcinomas (TNM stages III and IV), and in 27 of 47 patients (57.4%) with early-stage carcinomas (TNM stages I and II). A total of 23 (38.3%) lung ADC patients experienced metastasis. The patients with positive FGF4 expression had a higher rate of metastasis (19/38, 50.0%) than those with negative FGF4 expression (4/22, 18.2%). No correlations were found between FGF4 expression level and patient age or gender, tumor size or location. Interestingly, we observed differences in FGF4 expression within tumors, with strong FGF4 expression observed in tumor cells located close to stroma (Figure 6A). We also analyzed FGF4 expression in 21 sets of matched specimens (including lung ADC primary foci and lymph node metastatic foci) obtained from one patient to gain a better picture of FGF4 expression in the lung ADC progression. Table 2 and Figure 6B show that FGF4 expression in the lymph node metastatic foci was higher than in the primary foci (*P* < 0.05), implying that FGF4 may be involved in the EMT process of lung ADC cells.

**Table 1:** Correlation between FGF4 and clinicopathologic characteristics of lung adenocarcinoma

Variable	Total(%)	FGF4 expression			χ2	*P* value
		−	+	++		
Age						
<50	7 (11.7)	4 (57.1)	1(14.3)	2(28.6)	3.021	0.221
≥50	53 (88.3)	18 (34]	26(49.1)	9 (17)		
Sex						
Male	26 (43.3)	10 (38.5)	9 (34.6)	7(26.9)	2.986	0.225
Female	34 (56.7)	12 (35.3)	18 (52.9)	4(11.8)		
Lobe of primary tumor						
Upper	37(61.7)	14(37.8)	16(43.2)	7(18.9)	0.120	0.942
Middle/Lower	23(38.3)	8(34.8)	11(47.8)	4(17.4)		
Tumor size(cm)						
≥3	28 (46.7)	9 (31.2)	14 (50)	5(17.9)	0.591	0.744
<3	32 (53.3)	13 (40.6)	13(40.6)	6(18.8)		
Histological type						
Lepidic predominant	21 (35.0)	12 (57.1)	7 (33.3)	2(9.5)	15.952	0.043*
Acinar predominant	17 (28.3)	4(23.5)	12(70.6)	1(5.9)		
Solid predominant	10 (16.6)	3(30.0)	4(40.0)	3(30.0)		
Micropapillary predominant	8(13.3)	1(12.5)	3(37.5)	4(50.0)		
Papillary predominant	4(6.7)	2(50.0)	1(25.0)	1(25.0)		
TNM stage						
TNM I	33(55.0)	18(54.5)	11(33.3)	4(12.1)	17.197	0.009*
TNM II	14(23.3)	2(14.3)	10(71.4)	2(14.3)		
TNM III	7(11.7)	1(14.3)	2(28.6)	4(57.1)		
TNM IV	6(10.0)	1(16.7)	4(66.7)	1(16.7)		
Metastasis						
Present	23(38.3)	4 (17.4)	13(56.5)	6(26.1)	6.103	0.047*
Absent	37(61.7)	18 (48.6)	14(37.8)	5(13.5)		

* Significantly different.

**Table 2:** FGF4 expression in matched lung adenocarcinoma primary foci and metastatic foci in lymph nodes

	FGF4 expression n(%)				χ2	*P* value
	Total	−	+	++		
Primary foci	21	10(47.6)	9(42.9)	2(9.5)	6.371	0.041*
Metastatic foci	21	5(23.8)	7(33.3)	9(42.9)		
Total	42	15(35.7)	16(38.1)	11(26.2)		

* Significantly different.

### Expression of FGF4 is concomitant with EMT immunohistochemical features and Orai1 expression

**Figure 7:**
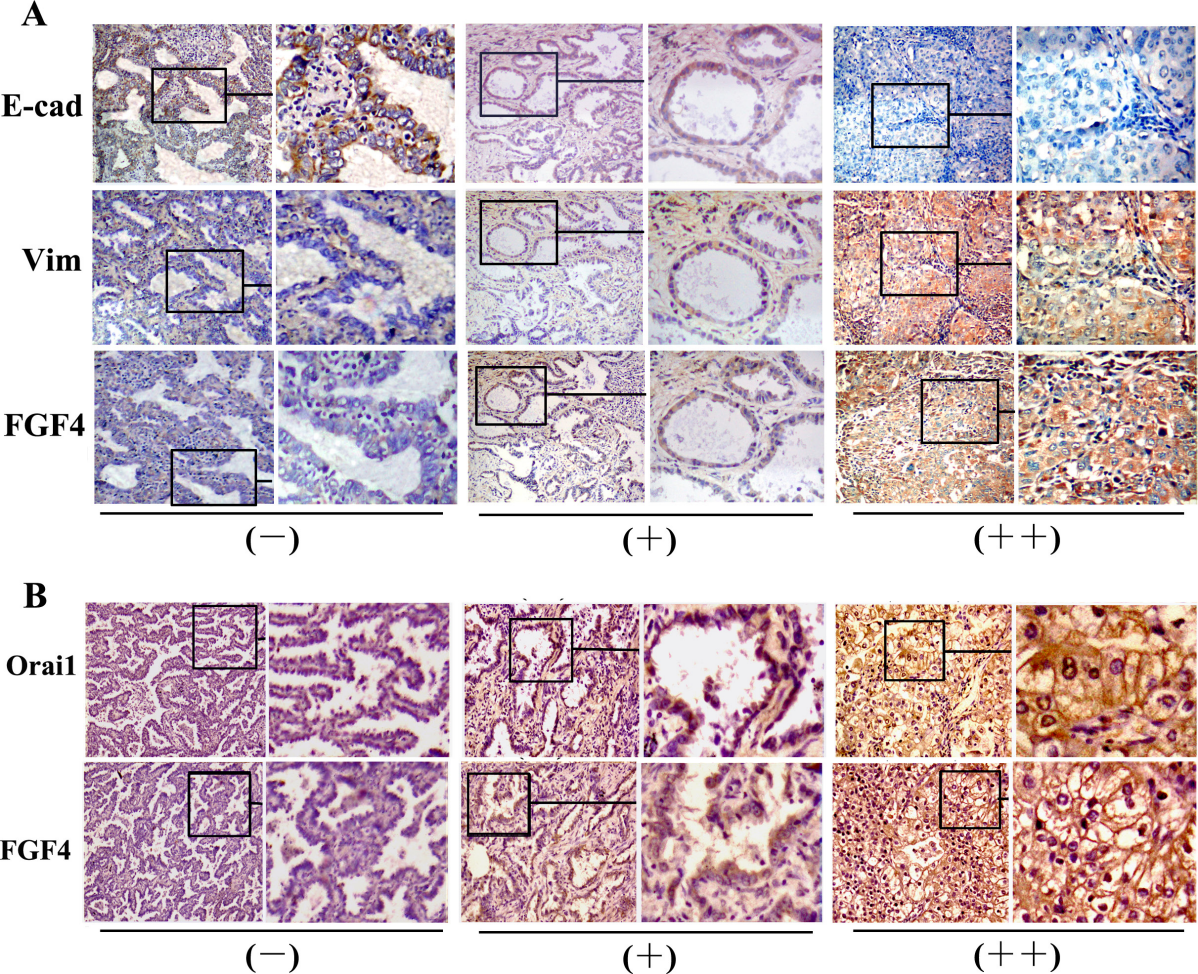
Expression of FGF4 is concomitant with EMT immunohistochemical features and Orai1 expression **A.** Immunohistochemical staining of E-cadherin, Vimentin, and **B.** Orai1 in the three groups with different FGF4 levels (−,+,++) in human lung ADC tissue samples (200×).

To further assess the relationship between FGF4 and EMT in human lung ADC tissues, we investigated the expression of E-cadherin and Vimentin. As shown in Table 3 and Figure 7A, the FGF4-negative group showed higher E-cadherin expression (*P* < 0.05) and lower Vimentin expression (*P* < 0.05) than the weakly and strongly positive groups, thereby confirming the EMT-promoting effect of FGF4 on lung ADC.

**Table 3:** Correlation between expression of FGF4 and epithelial-mesenchymal transition-associated proteins

Variable	Total(%)	FGF4 expression			χ2	*P* value
		−	+	++		
E-cadherin expression						
-	10(16.7)	1(10.0)	4(40.0)	5(50.0)	8.958	0.011*
+	50(83.3)	21(42.0)	23(46.0)	6(12.0)		
Vimentin expression						
-	52(86.7)	21(40.4)	25(48.1)	6(11.5)	12.112	0.002*
+	8(13.3)	1(12.5)	2(25.0)	5(62.5)		

* Significantly different.

FGF4 and Orai1 expression in the 60 specimens was analyzed to assess the relationship between FGF4 and SOCE. Orai1 expression was higher (*P* < 0.05) in the samples with weakly and strongly positive FGF4 expression than in those with negative FGF4 expression (Table 4 and Figure 7B). These findings suggested that FGF4 may induce EMT by elevating SOCE.

**Table 4:** Correlation between expression of FGF4 and Orai1

Variable	Total(%)	FGF4 expression			χ2	*P* value
		−	+	++		
Orai1 expression					12.482	0.014*
-	25(41.7)	15(60)	6(24)	4(16)		
+	30(50)	6(20)	9(63.3)	5(16.7)		
++	5(8.3)	1(20)	2(40)	2(40)		

* Significantly different.

## DISCUSSION

Lung cancer is a leading cause of cancer-associated death worldwide. ADC accounts for almost 40% of all lung malignancies and its incidence has rapidly increased in recent years [12]. Several targeted drugs against growth factors or their receptor tyrosine kinases have been used to treat ADC [4, 11-13, 28-30]; however, the prognosis of patients remains dismal. A better understanding of the molecular mechanisms involved in the development and progression of lung ADC might facilitate the development of novel treatment strategies that improve patient prognosis.

FGF/FGFR signaling is involved in many physiological and pathological processes. In general, FGFRs encoding exon IIIb (FGFR-IIIb) are expressed in epithelial cells, whereas FGFRs encoding exon IIIc (FGFR-IIIc) are expressed in mesenchymal cells [7, 14, 16, 31]. In EMT, FGFR2-IIIb switches to FGFR2-IIIc. FGF4, which binds FGFR2-IIIc with high affinity, was previously reported to be highly expressed in advanced-stage and poor-prognosis cases of serous ovarian cancer [32]. FGF7, which exerts its biological effects by binding to FGFR-IIIb, was observed to promote tumor angiogenesis and migration of pancreatic cancer [33]. In this study, we found that FGF4, but not FGF7, could downregulate E-cadherin expression and upregulate the expression of Vimentin and the two EMT transcription factors Snail and Twist. We also demonstrated that FGF4 treatment could cause a switch from FGFR2-IIIb to FGFR2-IIIc. Except for altering the expression of differentiation proteins, FGF4 can induce cancer cells to be more mesenchymal-like and enhance their proliferation, invasion/migration, and colony initiation abilities. Experiments conducted on human lung ADC tissue samples showed that FGF4 expression was relatively high in lymph-node metastatic foci and in lung ADC tissues with metastasis. Mouse experiments also indicated that FGF4 treatment may induce tumor metastasis. Immunohistochemical staining of human and mice tissue samples demonstrated that FGF4 could induce a phenotype displaying EMT. These results were consistent with a previous study, in which FGF4 was found to maintain trophoblast stem cells under undifferentiating, self-renewing conditions and display decreased E-cadherin expression and increased Slug and Twist expression [34].

Following FGF binding and receptor dimerization, downstream components including AKT and ERK kinases are activated to initiate several signaling pathways. FGF/FGFR signaling also confers selective and strong binding to phospholipase Cc for the catalysis of phosphatidylinositol diphosphate to generate two effectors, namely, diacylglycerol and IP3 [1, 2, 17, 19]. IP3 then binds to the IP3 receptor on the endoplasmic reticulum membrane and evokes Ca^2+^ release from calcium stores into the cytosol. Once the STIM protein senses a Ca^2+^ level drop in the endoplasmic reticulum, it binds with Orai channels and elicits Ca^2+^ influx. This process is known as SOCE [19, 25, 26, 35]. In this study, we observed that the expression of phosphorylated AKT and ERK in FGF4-stimulated cells were not increased compared with control cells. On the other hand, FGF4 treatment remarkably upregulated Orai1 expression and elevated the intracellular calcium concentration. In the human lung ADC tissue samples, we observed higher levels of Orai1 expression in the FGF4 strong expression group than in the weak and negative expression groups. A similar result was observed in the mouse xenograft model. This suggests that FGF4 may exert its EMT-promoting effect by elevating SOCE and not through the AKT and ERK signaling pathways.

We also showed that either SOCE pharmacological inhibitor BHQ or Orai1 knockdown could abrogate the induction of the EMT response to FGF4 *in vitro*. Furthermore, BHQ could restrained tumor formation and metastasis in an mouse xenograft model. All of these results indicated that FGF4-induced EMT correlates with enhancement of SOCE. SOCE promotes malignant phenotypes [19] and reduces the production of VEGF, tissue factor and COX-2 (which are essential for angiogenesis) in cancer cells [26, 36]. Aberrant SOCE induces directional migration and inhibition of SOCE results in the upregulation of p21 and the downregulation of Cdc25C, cyclinE, cyclinD, CDCK2, and CDCK4, which eventually elicit cell-cycle arrest [37]. The clinical diagnostic and prognostic potential of Orai1 and STIM1 have also been demonstrated in many cancer types, including prostate cancer, melanoma, NSCLC, and colorectal cancer [19, 26].

SOCE has been reported to be involved in TGF-β or EGF-induced EMT in breast cancer cells [27, 21]. Similarly, here we report that SOCE helps to drive the FGF4-induced EMT program. However, inconsistent results have been reported. A study by Niu *et al.* showed that FGF7 stimulates cell proliferation in pancreatic ductal epithelial cells [38]. According to previous reports, FGFs exert different actions depending on the type and origin of cells examined [39]. Future studies assessing the precise function of FGFs and their receptors in the EMT and tumor progression are needed. On the other hand, our results contradict a recent study by Davis *et al.*, who demonstrated that EGF-induced EMT in MDA-MB-468 breast cancer cells was associated with reduced SOCE [40]. In addition to FGF4/FGFR signaling, several other signaling pathways (*e.g.*, TGFβ, EGF, Notch, Wnt) have also been shown to induce EMT [5, 22, 41, 42]. Interestingly, some of these pathways are found among the functional gene sets associated with FGF/FGFR signaling and SOCE regulation. Future investigations may reveal how SOCE changes in EMT induced by alternative cytokines and signaling pathways.

## MATERIALS AND METHODS

### Cell culture and growth factor stimulation

Human lung ADC cell lines A549 and H1299 were obtained from the Cell Resource Center, Institute of Basic Medical Sciences, Chinese Academy of Medical Sciences, Peking Union Medical College (Beijing, China). Both cell lines were cultured in recommended medium (RPMI-1640) supplemented with 10% fetal bovine serum (Hyclone, Logan, Utah, USA) at 37 °C in 5% CO_2_ incubator. Sometimes, 10% fetal bovine serum was changed to serum-starved condition depending on the experiment. After 24 h of starvation in serum-free medium, cells were treated with FGF4 and FGF7 (PeproTech Rocky Hill, NJ, USA).

### Antibodies and reagents

The rabbit anti-Orai1, rabbit anti-Snail, rabbit anti-Vimentin and mouse anti-Ki-67 were obtained from Santa Cruz Biotechnology (Santa Cruz, CA, USA). Rabbit anti-Twist, mouse anti-Vimentin, rabbit anti-AKT, rabbit anti-p-AKT, rabbit anti-ERK and rabbit anti-p-ERK were obtained from Cell Signaling Technology (Beverly, MA, USA). Rabbit anti-Slug and rabbit anti-E-cadherin were from Abcam (Cambridge, UK). Mouse anti-β-actin was obtained from Tianjin Sungene Biotech Co, Ltd. BGJ398 was obtained from Selleck Chemicals (Houston, TX, USA). 2,5-di-tert-butylhydroquinone (BHQ) was obtained from Sigma (St. Louis, MO, USA).

### Western blot analysis

Cell lysates were prepared using RIPA lysis buffer. Protein (30-50 μg/lane) was separated by SDS-PAGE and transferred to polyvinylidene difluoride membranes. Subsequently, the membranes were incubated overnight with primary antibodies (E-cadherin 1:5000, Vimentin 1:1000, Snail 1:500, Slug 1:500, Twist 1:500, Orai1 1:500, AKT 1:1000, p-AKT 1:1000, ERK 1:1000, p-ERK1:1000 and β-actin 1:5000) at 4 °C. Blots were washed in TBS containing 0.1% Tween 20 and labeled with goat anti-mouse IgG-HRP or goat anti-rabbit IgG-HRP (1:5000; Santa Cruz Biotechnology). Equal loading of samples was confirmed by probing the membranes with β-actin antibody.

### RNA isolation, cDNA synthesis and RT-PCR analysis

RNA was isolated from cells using the RNeasy mini kit (QIAGEN Hilden, Germany) or TRIzol reagent (Invitrogen Carlsbad, CA, USA). Reverse transcription was performed with high capacity cDNA reverse transcription kits (Takara Biotechnology, Shiga, Japan) according to the manufacturer's instructions. RT-PCR (98 °C for 5 min, 34 cycles at 98 °C for 30 s, 55 °C for 30 s, and 72 °C for 30 s, with an extension step of 10 min at 72 °C at the end of the last cycle) was carried out using 2×PCR Solution Premix Taq (R004A, Takara Biotechnology Shiga, Japan). PCR products were run on 1% agarose gel. The primers *FGFR2IIIb*: forward: 5′-*GCA CTC GGG GAT AAA TAG TTC* −3′; reverse: 5′-*TGT TTT GGC AGG ACA GTG AGC*-3′; *FGFR2IIIc*: forward: 5′-*GTT AAC ACC ACG GAC AAA GAG*-3′; reverse: 5′-*GGC GCT GGC AGA ACT GTC AAC* −3′; *FGF4*: forward: 5′-*ATG TCG GGG CCC GGG ACG GCC* −3′; reverse: 5′-*TCA CAG CCT GGG GAG GAA GTG* −3′; *FGF7*: forward: 5′-*ATG CAC AAA TGG ATA CTG ACA* −3′; reverse: 5′-*TTA TTG CCA TAG GAA GAA AGT* −3′.

### Small interfering RNAs and transfections

The Orai1 gene-specific short interfering (si-1: *CCTTCGGCCTGATCTTTAT* si-2: *GCAACGTGC ACAATCTCAA* si-3: *GCTCACTGGTTAGCCATAA*), and non-specific control siRNA were purchased from RiboBio (Shanghai, China). A549 or H1299 cells were transiently transfected using Fugene (Life Technologies, Carlsbad, CA, USA) according to the manufacturer's instructions. There were 3 specific siRNAs and protein expression levels were detected by western blot to evaluate the efficiency of knockdown after 24 h transfection.

### Phalloidin staining of F-actin

Cells were cultured on sterile glass cover slips and placed in serum-free medium transfected with FGF4/si-Orai1 or added with FGF4, FGF7 or FGF4/BHQ for 24 h before staining. Cells were fixed with ice-cold 4% paraformaldehyde for 10 min, quenched with 50 mmol/L NH_4_Cl for 5 min, permeabilized in 0.2% Triton X-100 for 10 min. The slips were incubated with the phalloidin conjugated to Alexa 594 for 40 min in the dark. Cells were washed five times with PBS and visualized with a confocal laser scanning microscopy (Leica TCS SP5, Leica Microsystems, Wetzlar, Germany).

### Cell proliferation assay

Cell proliferation was evaluated using the Cell Counting Kit-8 (CCK-8) assay (Dojindo Laboratories, Kumamoto, Japan). Cells (8×10^3^ cells/well) were seeded into 96-well flat-bottomed plates in 100 μL of complete medium. The cells were incubated overnight to allow for cell attachment and recovery and then added with or without FGF4, FGF7 for different periods (1, 2, 3, 4, or 5 days). In some experiments, cells were transfected with si-Orai1 or mixed with 50 μM BHQ. Before detection, cells were incubated with CCK-8 solution (10 μL) for 3 h. Absorbance was measured at 450 nm using a microplate reader according to the manufacturer's instruction.

### Migration/invasion assay

Cell motility was examined in a transwell assay using 24-well plates with uncoated inserts (8 μm pore, BD Biosciences San Jose, CA, USA) to examine migration or Matrigel-coated inserts to assess invasiveness. Briefly, 600 μL culture medium supplemented with FGF4 or FGF7 were added to the lower part of the chamber, whereas cells (1×10^5^ cells) in 200 μL culture medium were seeded to the upper part. For some experiments, cells were transfected with si-Orai1 or mixed with 50 μM BHQ. After incubation at 37 °C with 5% CO_2_ for 24 h, the passed cells were fixed and stained. The entire membrane was counted by light microscopy in six random fields.

### Soft agar colony formation assay

The agar was plated in 2 layers. A 0.6% agar dilution (Difco Agar Nobel) was placed on the bottom of a 6-well plate. Cells were seeded at a density of 1×10^4^/ml and mixed into the 0.3% agar top layer that had been prepared with or without FGF4 or FGF7. For some experiments, cells were transfected with si-Orai1 or added with 50 μM BHQ. Two weeks later, the cells were stained with 0.02% crystal violet, and photographed at 10 random fields in each well.

### Flow cytometry for intracellular calcium

Levels of intracellular calcium concentration were measured by flow cytometry. Cells were washed with Hank's balanced salt solution (HBSS) three times, loaded with 1 ml HBSS containing 5 μmol/L Fluo 3-AM (Dojindo, Japan) and incubated at 37 °C for 60 min. Cells were washed with HBSS three times and incubated with HBSS for 30 min. The cells were collected and resuspended in PBS. The intracellular calcium concentration in a population of 50,000 cells was measured using an Accuri C6 flow cytometer (BD Biosciences San Jose, CA, USA).

### Co-immunoprecipitation (Co-IP) assay

The cells were washed with PBS, lysed in ice-cold Co-IP lysis buffer and protease inhibitor cocktail, and were then incubated on ice for 30 min. The insoluble material was pelleted at 12,000 g for 15 min at 4 °C, pre-cleared by incubation with protein A/G PLUS-Agarose (Santa Cruz Biotechnology, Inc.) and the aliquots were co-immunoprecipitated with anti-FGF4 primary antibody or IgG followed by incubation with protein A/G PLUS-Agarose beads for a further 1 h at 4 °C. The immunoprecipitated complexes were washed with co-IP washing buffer [200 mM Tris (pH 7.4), 150 mM NaCl, 0.5% NP-40, and 1 mM EDTA] three times. Immunoprecipitated proteins were separated by 10% SDS-PAGE. Then western blot was performed as described above.

### Xenograft mouse model

Thirty mice (Wei Tong Li Hua Experimental company, Beijing, China) were randomly divided into three groups and received 3 × 10^6^ H1299 cells by subcutaneous injection in the right groin. In the FGF4 and FGF4/BHQ groups, the mice were given three subcutaneous injections of FGF4 (500 ng/mouse) per week. In the FGF4/BHQ group, the mice were given BHQ (1 mg/kg) three times per week after having been injected with FGF4 during fifteen days. All animal care and handling procedures were approved by the Institutional Animal Use and Care Committee of Tianjin Medical University. Tumor size was measured every 5 days for 30 days. Tumor volumes were calculated using the following formula: volume = (length×width^2^)/2. Tumor samples were formalin fixed, paraffin embedded and subjected to H&E and immunohistochemical staining.

### Hematoxylin-eosin staining

Tissues were fixed in 10% neutral buffered formalin for 24 h, embedded in paraffin wax, cut into 4 *μ*m thicknesses, deparaffinized in xylene, and processed with graded ethanol series. Sections were stained with Hematoxylin and Eosin and visualized using an Olympus BX51 microscope.

### Clinical samples

We collected 60 formalin-fixed, paraffin-embedded lung ADC tissue samples and 21 sets of matched lung ADC primary foci and metastatic foci from the Department of Pathology at Tianjin Medical University Cancer Institute and Hospital from January 2014 to December 2014. None of the patients had received any chemotherapy or radiotherapy before their operation. All the samples underwent a uniform protocol for fixation/dissection and processing schedule. All sections were evaluated by two senior pathologists. The use of the tissue samples in this study was approved by the Institutional Research Committee.

### Immunohistochemical staining

The paraffin-embedded sections were deparaffinized by sequential washing with xylene, graded ethanol and PBS. Endogenous peroxidase activity was blocked with 0.3% H_2_O_2_ for 30 min before antigen retrieval by microwave treatment in citrate phosphate buffer. The sections were incubated overnight at 4 °C with rabbit anti-FGF4, rabbit anti-Orai1, rabbit anti-E-cadherin, rabbit anti-Vimentin and mouse anti-Ki-67 diluted in PBS (FGF4 1:50, Orai1 1:50, E-cadherin: 1:50, Vimentin: 1:50, Ki-67: 1:100). As a control, sections were stained with the biotinylated anti-rabbit only. After being washed three times with PBS, the sections were incubated with appropriate secondary antibodies for 1 h at room temperature. The processed sections were examined using an Olympus BX51 microscope images were captured using the AnalySIS program.

The expression of FGF4, E-cadherin, Vimentin, and Orai1 was analyzed only histologically in neoplastic epithelial cells. FGF4 and Orai1 staining was considered immunoreactive when brown granules were identified in the cytoplasm. The staining intensity of FGF4 and Orai1 was graded on a scale from 0 to 2 (0 for no staining, 1 for weak immunoreactivity, 2 for strong immunoreactivity). Percentage immunoreactivity was scored on a scale from 0 to 3 (0 for no positive cells, 1 for < 25% of cells being positive, 2 for 25% to 50% of cells being positive, and 3 for > 50% of cells being positive). The two scores were multiplied to obtain a composite expression score. The final expression level was classified as negative (−) (score = 0,), weakly positive (+) (score = 1, 2, or 3), or strongly positive (++) (score =4, 5, or 6). E-cadherin expression was considered to be positive if > 90% of tumor cells exhibited a staining pattern similar to that in normal epithelial cells. Vimentin expression was classified as positive when > 10% tumor cells were stained. Ki-67 expression was considered to be positive if > 30% of tumor cells with brown stained nucleus.

### Statistical analysis

SPSS v.16.0 software (SPSS Inc., Chicago, IL, USA) was used for data analysis. Data are expressed through at least triplicate independent determinations. Data were presented as means ± SD. The associations between FGF4 and clinicopathologic parameters and the differential expression of E-cadherin, Vimentin and Orai1 between different FGF4 expression level groups were assessed with Fisher's exact test and chi-square test. Differences between groups were assessed by the Mann–Whitney U-test and the Student's t-test. Two-tailed values of *P* < 0.05 were considered statistically significant.

## SUPPLEMENTARY MATERIALS FIGURES


