# Supplementary material for: FGF4 induces epithelial-mesenchymal transition by inducing store-operated calcium entry in lung adenocarcinoma
Source: Oncotarget. 2016 Sep 22;7(45):74015–30. doi: 10.18632/oncotarget.12187 (PMC5342032; doi:10.18632/oncotarget.12187)
Supplement: Supplementary file 1 [file oncotarget-07-74015-s001.pdf]

FGF4 induces epithelial-mesenchymal transition by inducing store-operated calcium entry in lung adenocarcinoma

SUPPLEMENTARY FIGURES

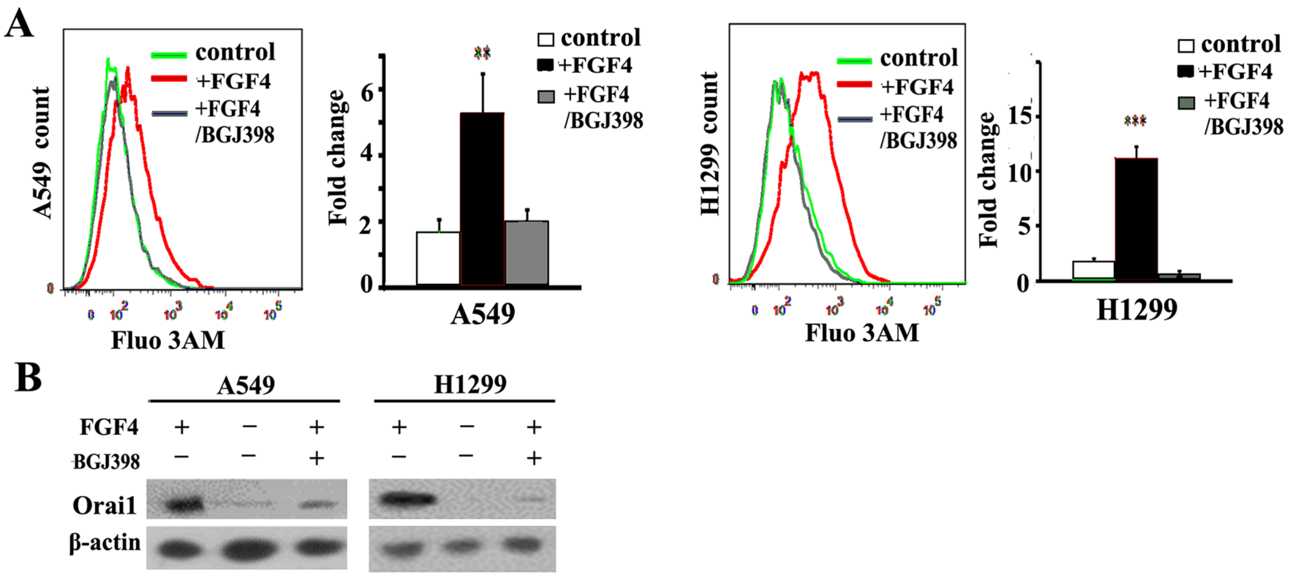

Supplementary Figure S1: FGFR inhibitor NVP-BGJ398 alleviates intracellular calcium concentration and abolishes the expression of Orai1 caused by FGF4 stimulation in A549 and H1299 cells. A. Level of intracellular Ca^{2+} using Fluo 3-AM measured by flow cytometry, and **B.** western blot to measure Orai1 expression in A549 and H1299 cells 5 h after NVP-BGJ398 treatment and subsequent stimulation with FGF4 for 24 h. All graphs represent the mean ± SD of three independent experiments. The axis represents the fold change in the number of cells. $^{**}P < 0.01$, $^{***}P < 0.001$.

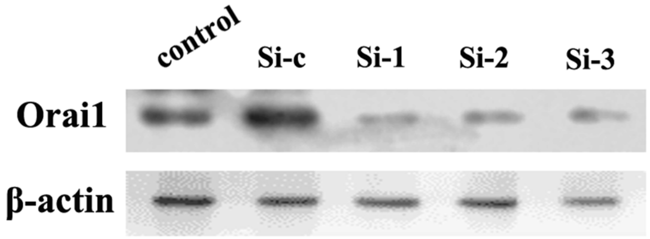

Supplementary Figure S2: Western blot of Orai1 in A549 and H1299 cells transfected with Orai1 siRNA(si-1,si-2,si-3).

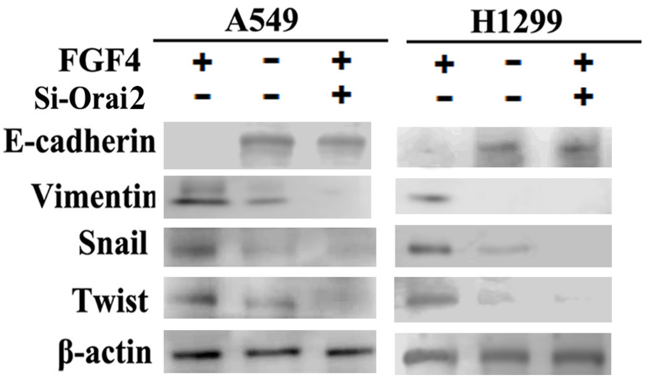

Supplementary Figure S3: Western blot of EMT-associated proteins (E-cadherin, Vimentin, Snail and Twist) in A549 and H1299 cells transfected with Orai1 siRNA (si-2).
